# Computational fluid dynamics analysis of middle ear pressure dynamics: Evidence for efficient pressure equalization during partial eustachian tube opening

**DOI:** 10.1371/journal.pone.0344128

**Published:** 2026-03-10

**Authors:** Yanzhuo Zhang, Fang Guo, Hua Liang, Yuetang Wang, Lan Su, Xin Yang, Ranran Liu, Xin-ge Lu, Yongtao Tian, Chunhua Wang, Jin Wang

**Affiliations:** 1 Department of Otorhinolaryngology, Hebei Provincial Eye Hospital, Hebei Provincial Key Laboratory of Ophthalmology, Hebei Provincial Eye Institute, Xingtai, China; 2 Department of Radiology, Hebei Provincial Eye Hospital, Hebei Provincial Key Laboratory of Ophthalmology, Hebei Provincial Eye Institute, Xingtai, China; 3 Department of Intensive Care Medicine, Handan Central Hospital, Handan, China; 4 School of Energy and Environmental Engineering, Hebei University of Technology, Tianjin, China; NED University of Engineering and Technology, PAKISTAN

## Abstract

**Background:**

Eustachian tube (ET) dysfunction is associated with middle ear pathologies; however, the quantitative relationship between ET opening and pressure equalization remains insufficiently characterized. Computational fluid dynamics (CFD) offers a robust tool for analyzing middle ear pressure dynamics, particularly in elucidating pressure equilibrium mechanisms under partial ET opening conditions.

**Objective:**

This study aimed to investigate pressure dynamics in the tympanic cavity, mastoid antrum, and air cells during ET opening using CFD, to compare pressure distributions between full and partial openings, and to determine whether partial opening can achieve equilibration equivalent to full opening.

**Methods:**

Eight normal temporal bones were reconstructed from high-resolution computed tomography scans of four healthy adults. ET openings were simulated at 10%, 30%, 50%, and 100% patency using CFD, and results were validated against in vivo Tubomanometry data. Pressure variations in the tympanic cavity, mastoid antrum, and air cells were monitored throughout the process. Mesh independence was verified to ensure reliability, and statistical analyses were conducted using SPSS 27.0, with *P* < 0.05 considered significant.

**Results:**

CFD simulations revealed distinct pressure dynamics within the ET–middle ear system. Airflow velocity peaked at the narrow isthmus, generating a localized pressure drop. Effective middle ear pressure equilibration—across the tympanic cavity, antrum, and mastoid air cells—was achieved with partial ET opening in most cases: 30% opening sufficed for full equilibration in two ears, while 50% opening achieved complete equilibration in six. This equivalence to full patency was consistently observed during pressurization, stabilization, and depressurization phases.

**Conclusion:**

Effective middle ear pressure equilibration can be achieved with partial ET opening (50%) in most cases (75% of ears). These findings provide valuable insight into middle ear physiology and its response under pathological conditions, offering a theoretical basis for optimizing the management of ET dysfunction.

## Introduction

The Eustachian tube (ET) is a collapsible passage connecting the nasopharynx to the middle ear. Its periodic opening plays a critical role in maintaining middle ear pressure equilibrium, preventing reflux and pathogen entry, and facilitating mucus drainage. ET dysfunction is a major predisposing factor for middle ear pathologies, including otitis media with effusion, tympanic membrane retraction, and cholesteatoma [1–3]. Shan et al. reported that the overall prevalence of Eustachian tube dysfunction (ETD) among adults in the United States was approximately 4.6% [3], while the prevalence in children was 6.1% [4]. ETD significantly affects hearing and quality of life. Therefore, understanding the pressure dynamics within the middle ear and mastoid air cell system during ET opening is essential for elucidating the pathophysiology of these disorders and improving clinical interventions.

Computational fluid dynamics (CFD), a technology capable of resolving complex fluid flow–related problems, has become an essential research tool across multiple scientific disciplines, including medicine. CFD provides a powerful method for investigating fluid flow and pressure variations within anatomical structures, overcoming the limitations of invasive experimental approaches in otology [5,6]. Early CFD studies on ET function primarily focused on anatomical modeling and airflow pattern characterization. Sheer et al. [5] used finite element analysis to simulate ET opening and luminal dilation in both adults and children, demonstrating the influence of anatomical variability on function. Subtil et al. [6] developed a CFD model of pediatric ears to examine airflow through the external auditory canal, tympanic cavity, and ET under submerged and non-submerged conditions, thereby providing insights into middle ear ventilation following tympanostomy tube placement. Other numerical and experimental studies have also attempted to explore middle ear pressure dynamics but have exhibited notable limitations.

Tang et al. [7] identified the ET isthmus as a key region for pressure drop through finite element modeling; however, their study lacked direct correlation with clinical pressure measurements. Notably, most previous research has adopted a binary “open/closed” perspective, overlooking the functional importance of partial opening. McDonald et al. [8] also demonstrated that complete ET patency is not required for ventilation, although the quantitative threshold for effective pressure equilibration remains undefined.

To address these gaps, the present study reconstructed anatomically accurate three-dimensional (3D) models of the ET and middle ear from high-resolution temporal bone computed tomography (HRCT) scans and simulated airflow characteristics under varying degrees of ET opening using CFD informed by ET pressure measurement data. This study provides the first quantitative evidence that effective pressure equilibration can occur with partial ET opening, offering new insights into middle ear physiology and the clinical management of ET dysfunction.

## Materials and methods

### Ethical approval and subject selection

This study was approved by the Medical Ethics Committee of Hebei Eye Hospital (Approval No. 2023KY32). Participant recruitment began on December 1, 2023, and ended on June 1, 2025. The English-language ethical review certificate (Approval No. 2025LW06), issued for this manuscript, serves as a supplementary document to the initial approval (No. 2023KY32), confirming that the study design and reported data are consistent with the content approved by the Medical Ethics Committee of Hebei Provincial Eye Hospital. All participants provided written informed consent before enrollment. No minors were included in this study; therefore, parental or guardian consent was not required. HRCT scans and Tubomanometry (TMM) tests were obtained from four healthy adult volunteers (eight ears; two males and two females; bilateral scanning) at Hebei Eye Hospital. Exclusion criteria included a history of middle ear disease, prior otologic surgery, congenital temporal bone anomalies, or any evidence of ET dysfunction based on audiometry or otoscopy.

### Tubomanometry test protocol

Tubomanometry (TMM) tests were performed at a pressure of 30 mbar. Because only one ear can be tested at a time, two pressure curves were obtained for each ear at each delivered pressure.

**Fig 1:**
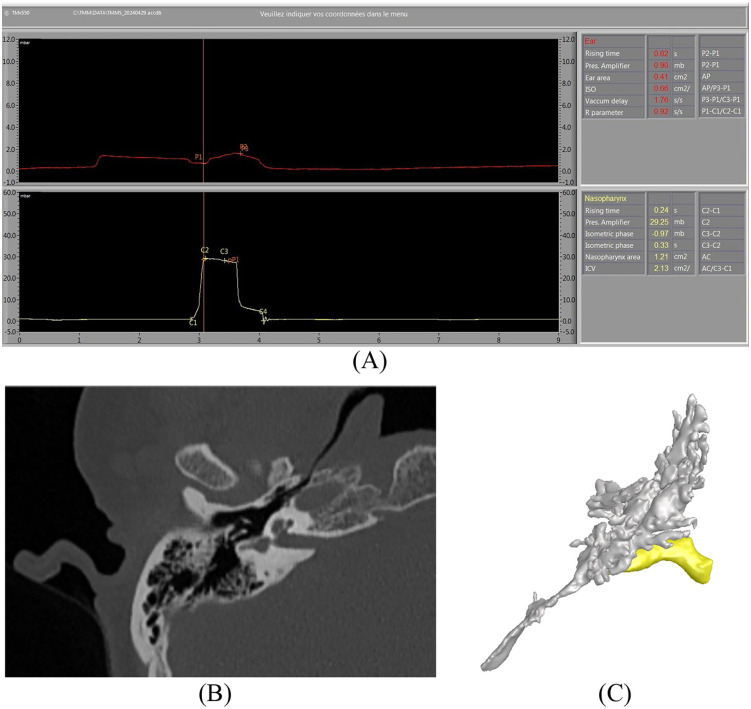
Tubomanometry and computed tomography (CT) imaging during Eustachian tube (ET) opening. (A) Representative Tubomanometry (TMM) tracing from a healthy volunteer demonstrating normal ET function. The examination was performed during swallowing at a nasal pressure of 30 mbar. C1 designates the onset of nasopharyngeal pressure elevation; C2 indicates the peak nasopharyngeal pressure and the beginning of the plateau phase; C3 marks the initiation of pressure descent and the end of the plateau phase; and C4 represents the completion of pressure descent. (B) ET imaging. Oblique-plane CT imaging performed during the Valsalva maneuver allows complete visualization of the radiolucent linear lumen of the ET. (C) CFD computational model. The computational domain includes the ET and middle ear cavity, while the external auditory canal was retained only for visualization. The outlet boundary was defined at the pharyngeal orifice of the ET. The isthmus region was segmented as an independent boundary, allowing parametric adjustment of its diameter to simulate varying degrees of patency: fully patent (100% tubal patency) and area-reduced configurations (10%, 30%, and 50% tubal patency).

The integrity of the tympanic membranes was confirmed before testing, and appropriately sized probes were selected. The ear probe was fitted and sealed into one external ear canal. Each participant was instructed to take a sip of water and hold it in their mouth. The two-pronged nasal probe was then positioned snugly over both nostrils, and the participant secured it in place using the thumb and index finger to ensure an airtight seal. The examiner closed the nasal probe valve, after which the participant was instructed to swallow. During velum closure, the tubomanometer detected an increase in nasal pressure and simultaneously delivered a secondary bolus of air to achieve the target nasal pressure of 30 mbar. The pressure curves generated from both the nasal cavity and external ear canal were recorded and digitized in time (X-axis) and pressure (Y-axis) dimensions (Fig 1A).

### CT scans and 3D geometry model reconstruction

The CT images (in-plane resolution of 512 × 512 pixels, pixel size 0.6 mm) were acquired using a 64-detector row CT scanner (Siemens). During scanning, participants were instructed to perform the Valsalva maneuver, and CT data were recorded during ET opening and exported in Digital Imaging and Communications in Medicine (DICOM) format. The open ET lumen was readily identifiable as a radiolucent area (Fig 1B). The DICOM images were imported into 3D Slicer (version 5.0.3) software for three-dimensional reconstruction of the ET and middle ear, and the reconstructed model was then exported in stereolithography (STL) format.

### CFD analysis

The CFD mesh was generated using ANSYS Fluent Meshing (version 17.0). The STL file was imported and repaired; faces were subsequently re-meshed and refined, and a hybrid volumetric mesh with polyhedral and prism-layer cells was generated.

**Fig 2:**
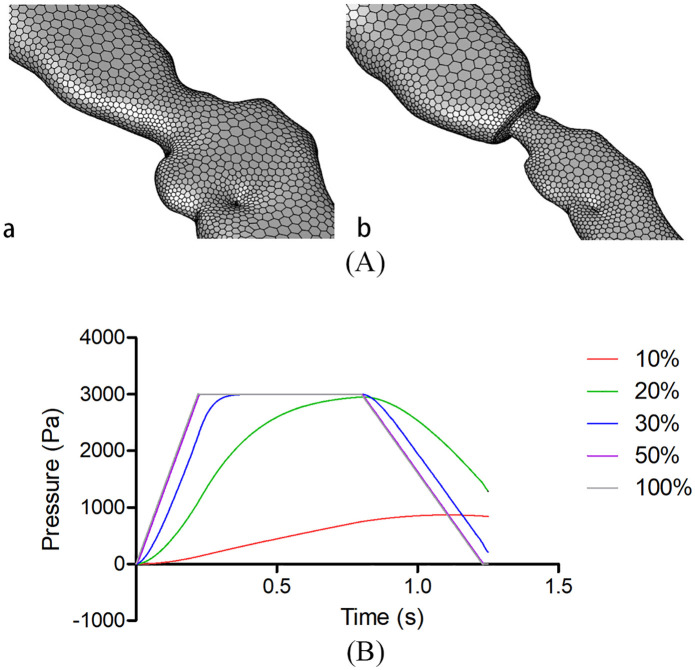
Eustachian tube (ET) patency configurations and pressure measurement results. (A) Computational meshes for the fully patent ET (left) and the 50% patency configuration (right). All other stenotic models followed the same meshing protocol as the 50% configuration. (B) Pressure curves under varying ET patency conditions. When ET patency was maximally restricted to 10%, middle ear pressure remained elevated after depressurization. Additional middle ear pressure measurements at 20% tubal patency were obtained from specimens showing comparable pressure recovery between partial (50%) and full (100%) patency states, revealing consistent trends across conditions.

As shown in Fig 1C, the computational domain included the ET and middle ear cavity, while the external auditory canal was retained solely for visualization. The outlet boundary was defined at the pharyngeal orifice of the ET. The isthmus region of the ET was segmented as an independent boundary, allowing parametric variation of diameters to simulate different degrees of patency: fully patent (100% tubal patency) and area-reduced configurations (10%, 30%, and 50% tubal patency). Four separate CFD models were developed for each ear to simulate these patency conditions. Fig 2A illustrates the fully patent and 50% patency configurations, with other cases following analogous treatment.

The CFD simulations were performed using ANSYS Fluent (version 17.0). Flow fields were computed by numerically solving the Navier–Stokes equations, discretized using the finite volume method.

The Realizable k–ε turbulence model was employed. Air was modeled as an ideal gas with an initial pressure of 1 atmosphere (P = 101,325 Pa). The pharyngeal orifice of the ET was defined as a pressure outlet, with a time-dependent pressure profile derived from TMM measurements: a linear ramp from 0 to 3000 Pa, maintained for a specified duration, and then linearly decreased.

All remaining surfaces were defined as no-slip, isothermal walls with a temperature of 36.5 °C. The pressure–velocity coupling was resolved using the SIMPLE algorithm, and the second-order upwind scheme was applied to discretize the pressure, momentum, turbulent kinetic energy (k), turbulent dissipation rate (ε), and energy equations. Three monitoring points were placed in the tympanic cavity, tympanic antrum, and mastoid air cells to record local pressure variations during transient simulation.

Mesh independence was validated using four progressively refined configurations, as presented in Table 1. Pressure values at the mastoid air cells from transient analysis at 0.1 s were compared. The results showed negligible discrepancies (<1.5%) between the fine and ultra-fine meshes. Therefore, the fine mesh configuration was selected for all subsequent CFD simulations to achieve an optimal balance between solution accuracy and computational efficiency.

**Table 1:** Mesh independence validation.

Mesh Size/Million	0.69(Coarse)	1.10(Medium)	1.75(Fine)	2.47(Ultra-fine)
Pressure/Pa	1341.70	1307.24	1257.18	1254.70

### Statistical analysis

Statistical analyses were performed using SPSS Statistics (version 27.0; IBM Corporation). Analysis of variance (ANOVA) was used to evaluate statistical significance, while the chi-square test or one-way ANOVA was applied for between-group comparisons. A significance level of *P* < 0.05 was considered statistically significant.

## Results

### CFD model validation with clinical data

To confirm the reliability of the CFD simulations, the simulated pressure curves were correlated with in vivo TMM data obtained from healthy participants.

To further validate model consistency under different functional conditions, representative pressure data were selected from six ET patency levels (0.1, 0.2, 0.3, 0.5, 0.7, and 1.0, corresponding to 10%, 20%, 30%, 50%, 70%, and 100% patency). Validation focused on the pressurization phase (C1–C2) and stabilization phase (C2–C3).

Pressurization phase (C1–C2): Across all patency levels, the CFD-simulated pressure rise trajectories closely matched the TMM-measured curves, with no statistically significant differences in key parameters.

Stabilization phase (C2–C3): Plateau pressure served as the primary indicator for evaluating pressure equilibration efficacy. The CFD-simulated plateau pressures were in strong agreement with both the TMM target pressure (30 mbar) and in vivo measurements, confirming the model’s capability to reproduce physiological pressure stabilization. During this phase, the CFD-predicted pressure remained constant, whereas the TMM-measured pressure demonstrated a slight downward trend. This difference can be attributed to several physiological factors. In TMM experiments, microscopic gaps may form between the nasopharyngeal or tympanic membrane probes and mucosal surfaces, causing gradual pressure leakage. Furthermore, transient patency fluctuations may occur in the human ET due to mucosal peristalsis and tensor veli palatini muscle activity, resulting in mild attenuation of pressure transmission. Additionally, continuous gas absorption by the middle ear mucosa can contribute to a minor pressure decrease. In contrast, the CFD model assumes a rigid, smooth, and completely sealed ET wall, eliminating random physiological variability; therefore, pressure within the computational domain strictly followed the defined boundary conditions.

It should be noted that the depressurization phase (C3–C4) was excluded from model validation for three main reasons:

1. Irrelevance to the core hypothesis: The central objective of this study was to determine whether partial ET patency can achieve effective pressure equilibration. This outcome depends on the dynamics of the pressurization phase (pressure rise) and the stabilization phase (plateau pressure). The depressurization phase, by contrast, only represents the rate of pressure release and does not influence the primary conclusion regarding equilibration.
2. Biologically reasonable model simplification: The CFD model assumes a rigid ET lumen—an approach consistent with prior studies [7]—and therefore excludes the effects of mucosal viscosity, mucus adhesion, and wall friction. In vivo, the flexible ET contracts after swallowing to accelerate pressure release, resulting in minor deviations from the rigid model. However, these differences are unrelated to the model’s capacity to simulate pressure equilibration efficacy.
3. Focus on key validation evidence: Excluding the nonessential depressurization phase prevents redundant data inclusion and ensures that the validation emphasizes the two critical phases—pressurization and stabilization—that directly support the study’s hypothesis.

### Pressure dynamics in the middle ear–ET system

**Fig 3:**
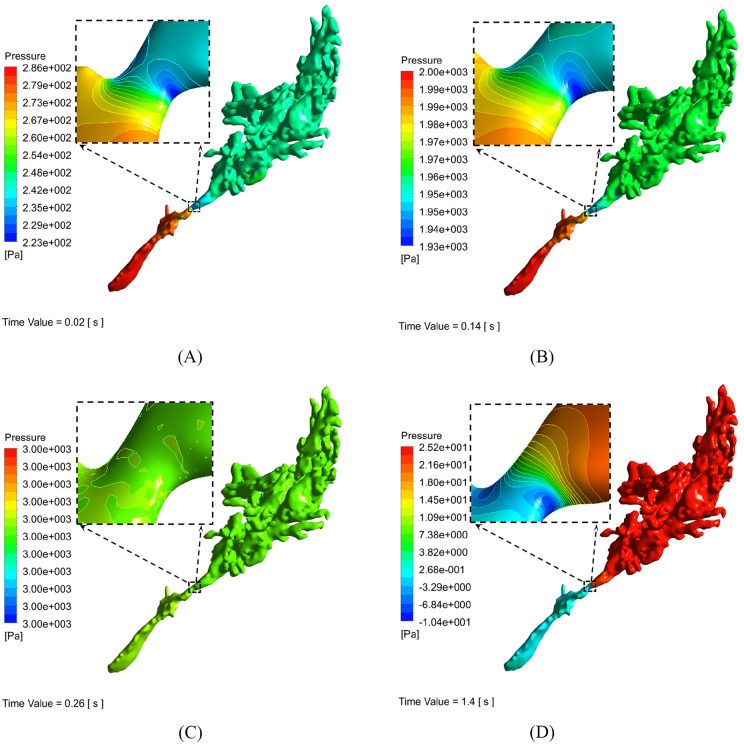
Pressure contour plots during the pressurization, stabilization, and depressurization phases (100% Eustachian tube [ET] patency). During the early pressurization phase (A: 0.02 s), continuous air injection drives airflow inward from the nasopharynx through the ET into the tympanic cavity, resulting in a gradual pressure increase within the tympanic cavity, mastoid antrum, and air cells (collectively referred to as the middle ear system). Magnified insets (dashed boxes) highlight the ET isthmus—the narrowest segment of the ET—where a localized low-pressure zone (cool colors) is visible, consistent with the Venturi effect. By the late pressurization phase (B: 0.14 s), airflow propagates further into the mastoid antrum and air cells, reducing the pressure gradient between the ET and middle ear structures, although the low-pressure region within the isthmus (cool colors in the inset) remains evident. In the stabilization phase (C: 0.26 s), pressure equalizes across the entire computational domain—including the ET, tympanic cavity, mastoid antrum, and air cells—with a uniform pressure distribution (depicted by consistent warm-to-green tones) and only minimal regional variation (inset). During the depressurization phase (D: 1.4 s), airflow reverses direction, moving outward from the tympanic cavity, mastoid antrum, and air cells through the ET toward the nasopharynx. The pressure gradually decreases along the ET pathway, eventually approaching atmospheric pressure over time. Color bars represent pressure magnitude (Pa), with warm colors denoting high pressure and cool colors denoting low pressure.

Under the applied pressure at the pharyngeal orifice, continuous pressurization drives airflow from the external environment through the ET into the middle ear, resulting in a gradual pressure increase throughout the computational domain. As the airflow passes through the isthmus—the narrowest cross-sectional region—its velocity reaches a maximum, while the pressure simultaneously drops to its lowest value. Beyond the isthmus, the pressure progressively rises toward the tympanic cavity. During the pressurization phase, the pressure within the ET remains consistently higher than that in the middle ear. In the stabilization phase, pressure equalizes across the entire computational domain, with only negligible differences. During the depressurization phase, airflow reverses direction (from the middle ear toward the nasopharynx), and the pressure gradually decreases from the middle ear to the ET, eventually approaching zero over time. Fig 3 presents the pressure contour plots corresponding to the pressurization, stabilization, and depressurization phases.

**Fig 4:**
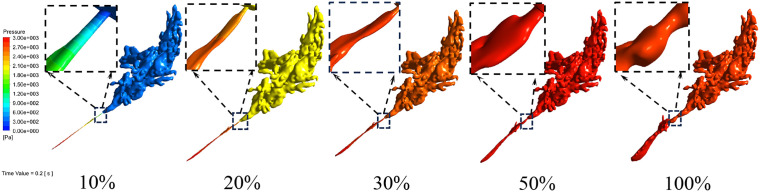
Pressure contour plots of the Eustachian tube (ET)–middle ear system under different ET patency levels during the late pressurization phase (0.2 s). This figure illustrates the spatial pressure distribution patterns of 10%, 20%, 30%, 50%, and 100% ET patency (from left to right) at the late pressurization phase (0.2 s). The dashed magnified boxes highlight the ET segment, representing the primary pressure transmission pathway, to clearly depict regional pressure variations. 10% patency: The ET and adjacent middle ear regions are dominated by cool colors, indicating pronounced low-pressure zones. 20% and 30% patency: Cool colors progressively transition to warm tones, and pressure gradients decrease as patency increases. 50% and 100% patency: The entire system displays uniform warm colors, reflecting consistent and equilibrated pressure distribution. Color bars represent pressure magnitude (Pa), where warm colors denote high-pressure zones and cool colors denote low-pressure zones.

To elucidate the spatial characteristics of pressure distribution under varying ET patency levels, pressure contour plots were generated for the late pressurization phase (0.2 s) (Fig 4). In conjunction with the quantitative pressure data presented in Tables 2–4, Fig 4 demonstrates a distinct pressure variation pattern. At 10% patency, the ET and adjacent middle ear structures displayed extensive cool color regions (indicating low-pressure zones), reflecting pronounced pressure gradients during transmission. This observation aligns with the elevated residual pressure noted in the 10% patency group (Fig 2B). As patency increased to 20% and 30%, the cool colors progressively transitioned to warmer tones, and the pressure gradients between the ET and middle ear regions markedly decreased. At 50% patency, the pressure contour pattern closely resembled that of 100% patency: the entire ET–middle ear system exhibited uniform warm colors (corresponding to high-pressure zones), signifying negligible pressure differences and unobstructed transmission. This spatial distribution finding further confirms that partial ET patency (≥50%) effectively eliminates pressure gradients within the system, thereby corroborating the quantitative results shown in Fig 2B and Tables 2–4, which indicate that 50% and 100% patency yield equivalent pressure equilibration efficacy.

**Table 2:** Comparison of pressure in the tympanic cavity among the area-reduced configuration groups during the pressurization, stabilization, and depressurization phases.

cases	10%			30%			50%			100%		
	C2-C1 (mbar)	C3-C2 (mbar)	C4-C3 (mbar)	C2-C1 (mbar)	C3-C2 (mbar)	C4-C3 (mbar)	C2-C1 (mbar)	C3-C2 (mbar)	C4-C3 (mbar)	C2-C1 (mbar)	C3-C2 (mbar)	C4-C3 (mbar)
1	2.74 ± 2.47	27.33 ± 4.82	22.18 ± 7.03	14.06 ± 8.64	30.00 ± 0.04	13.89 ± 9.70	14.84 ± 8.67	30.00 ± 0.01	13.13 ± 9.56	15.02 ± 8.67	30.00 ± 0.00	13.02 ± 9.53
2	4.38 ± 3.62	26.65 ± 4.57	20.46 ± 7.45	14.17 ± 8.64	30.00 ± 0.01	12.24 ± 10.12	14.86 ± 8.67	30.00 ± 0.01	11.64 ± 9.94	12.61 ± 8.85	30.00 ± 0.00	11.50 ± 9.62
*P* value	*P <* 0.05	*P <* 0.05	*P <* 0.05	NS	NS	NS	NS	NS	NS	－	－	－
3	1.09 ± 0.93	8.62 ± 2.96	14.76 ± 0.57	13.06 ± 8.41	30.00 ± 0.01	10.74 ± 6.97	14.81 ± 8.67	30.00 ± 0.01	14.01 ± 9.19	15.06 ± 8.68	30.00 ± 0.01	13.93 ± 9.19
4	0.48 ± 0.41	3.45 ± 1.16	7.28 ± 0.70	9.54 ± 6.87	29.08 ± 1.73	14.88 ± 8.84	13.98 ± 8.60	30.00 ± 0.00	14.73 ± 8.83	14.95 ± 8.67	30.00 ± 0.01	16.15 ± 8.70
5	1.28 ± 1.10	10.81 ± 3.73	17.63 ± 1.35	13.91 ± 8.61	29.98 ± 0.09	15.25 ± 8.65	15.41 ± 8.36	30.00 ± 0.01	14.44 ± 8.99	14.98 ± 8.67	30.00 ± 0.01	14.32 ± 8.99
6	10.20 ± 0.88	10.32 ± 3.85	16.87 ± 0.79	12.32 ± 8.30	29.94 ± 0.30	15.52 ± 9.15	14.58 ± 8.67	30.00 ± 0.01	13.82 ± 9.35	14.93 ± 8.67	30.00 ± 0.02	13.64 ± 9.33
7	0.91 ± 0.77	9.40 ± 3.58	15.45 ± 0.28	13.54 ± 8.53	29.97 ± 0.14	15.02 ± 0.13	14.87 ± 8.67	30.00 ± 0.00	14.44 ± 8.99	15.04 ± 8.67	30.00 ± 0.00	14.37 ± 8.98
8	0.44 ± 0.39	4.49 ± 1.78	8.34 ± 0.32	9.38 ± 6.90	29.51 ± 1.32	17.50 ± 8.52	14.31 ± 8.64	30.00 ± 0.00	14.60 ± 9.03	15.00 ± 8.67	30.00 ± 0.01	14.32 ± 9.01
*P* value	*P <* 0.05	*P <* 0.05	*P <* 0.05	*P <* 0.05	*P <* 0.05	*P <* 0.05	NS	NS	NS	－	－	－

NS: not statistically significant (*P* > 0.05).

**Table 3:** Comparison of pressure in the tympanic antrum among the area-reduced configuration groups during the pressurization, stabilization, and depressurization phases.

cases	10%			30%			50%			100%		
	C2-C1 (mbar)	C3-C2 (mbar)	C4-C3 (mbar)	C2-C1 (mbar)	C3-C2 (mbar)	C4-C3 (mbar)	C2-C1 (mbar)	C3-C2 (mbar)	C4-C3 (mbar)	C2-C1 (mbar)	C3-C2 (mbar)	C4-C3 (mbar)
1	2.73 ± 2.47	26.58 ± 5.22	24.05 ± 6.98	14.05 ± 8.64	30.00 ± 0.04	16.13 ± 10.59	14.84 ± 8.67	30.00 ± 0.01	17.18 ± 11.02	15.02 ± 8.67	30.00 ± 0.01	17.10 ± 11.03
2	4.38 ± 3.62	26.65 ± 4.57	20.46 ± 7.45	14.16 ± 8.64	30.00 ± 0.01	12.24 ± 10.12	14.86 ± 8.67	30.00 ± 0.01	11.64 ± 9.94	15.03 ± 8.67	30.00 ± 0.01	11.50 ± 9.87
P value	P < 0.05	P < 0.05	P < 0.05	NS	NS	NS	NS	NS	NS	－	－	－
3	1.10 ± 0.94	8.62 ± 2.96	14.89 ± 0.76	13.06 ± 8.41	29.94 ± 0.25	14.17 ± 9.28	14.81 ± 8.67	30.00 ± 0.01	13.29 ± 9.48	15.06 ± 8.68	30.00 ± 0.01	13.23 ± 9.47
4	0.48 ± 0.41	3.45 ± 1.17	7.29 ± 0.70	9.54 ± 6.88	29.08 ± 1.73	16.15 ± 8.70	13.98 ± 8.60	30.00 ± 0.02	14.88 ± 8.84	14.86 ± 8.67	30.00 ± 0.01	14.69 ± 8.83
5	1.27 ± 1.10	10.81 ± 3.73	17.63 ± 1.35	13.91 ± 8.61	29.98 ± 0.09	15.24 ± 8.65	15.39 ± 8.33	30.00 ± 0.01	14.44 ± 8.99	15.25 ± 8.50	30.00 ± 0.01	14.32 ± 8.99
6	10.20 ± 0.88	10.32 ± 3.85	16.87 ± 0.79	12.33 ± 8.31	29.94 ± 0.30	15.53 ± 9.16	14.58 ± 8.67	30.00 ± 0.01	13.82 ± 9.35	14.93 ± 8.67	30.00 ± 0.02	13.64 ± 9.33
7	0.92 ± 0.77	9.40 ± 3.59	15.45 ± 0.29	13.55 ± 8.54	29.97 ± 0.15	15.02 ± 8.98	14.87 ± 8.67	30.00 ± 0.00	14.44 ± 8.99	15.04 ± 8.67	30.00 ± 0.00	14.37 ± 8.98
8	0.45 ± 0.39	4.49 ± 1.78	8.34 ± 0.33	9.39 ± 6.90	29.25 ± 1.58	21.35 ± 9.14	14.31 ± 8.64	30.00 ± 0.01	19.34 ± 10.34	15.00 ± 8.67	30.00 ± 0.01	19.14 ± 10.43
P value	P < 0.05	P < 0.05	P < 0.05	P < 0.05	P < 0.05	P < 0.05	NS	NS	NS	－	－	－

NS: not statistically significant (*P* > 0.05).

**Table 4:** Comparison of pressure in the air cells among the area-reduced configuration groups during the pressurization, stabilization, and depressurization phases.

cases	10%			30%			50%			100%		
	C2-C1 (mbar)	C3-C2 (mbar)	C4-C3 (mbar)	C2-C1 (mbar)	C3-C2 (mbar)	C4-C3 (mbar)	C2-C1 (mbar)	C3-C2 (mbar)	C4-C3 (mbar)	C2-C1 (mbar)	C3-C2 (mbar)	C4-C3 (mbar)
1	2.75 ± 2.48	27.33 ± 4.82	22.18 ± 7.03	14.06 ± 8.64	30.00 ± 0.03	14.16 ± 9.58	14.84 ± 8.67	30.00 ± 0.01	13.12 ± 9.56	15.02 ± 8.67	30.00 ± 0.01	13.02 ± 9.53
2	4.38 ± 3.62	26.65 ± 4.57	20.46 ± 7.45	14.16 ± 8.64	30.00 ± 0.03	12.24 ± 10.12	14.86 ± 8.67	30.00 ± 0.01	11.64 ± 9.94	15.03 ± 8.67	30.00 ± 0.01	11.50 ± 9.89
*P* value	*P <* 0.05	*P <* 0.05	*P <* 0.05	NS	NS	NS	NS	NS	NS	－	－	－
3	1.09 ± 0.93	8.79 ± 3.04	15.72 ± 0.99	12.99 ± 8.46	29.94 ± 0.24	14.09 ± 9.36	14.76 ± 8.67	30.00 ± 0.01	13.30 ± 9.48	15.01 ± 8.67	30.00 ± 0.01	13.23 ± 9.47
4	0.48 ± 0.41	3.45 ± 1.17	7.29 ± 0.70	9.56 ± 6.87	29.08 ± 1.73	16.16 ± 8.70	14.07 ± 8.57	30.00 ± 0.01	14.88 ± 8.84	14.51 ± 8.37	30.00 ± 0.01	14.69 ± 8.83
5	1.28 ± 1.10	10.88 ± 4.16	17.67 ± 1.40	13.91 ± 8.61	29.98 ± 0.10	15.42 ± 8.65	15.42 ± 8.36	30.00 ± 0.01	14.44 ± 8.99	14.98 ± 8.66	30.00 ± 0.01	14.32 ± 8.99
6	10.20 ± 0.88	10.32 ± 3.85	16.87 ± 1.79	12.33 ± 8.31	29.94 ± 0.30	15.53 ± 9.16	14.58 ± 8.67	30.00 ± 0.02	13.82 ± 9.35	14.93 ± 8.67	30.00 ± 0.02	13.64 ± 9.33
7	0.92 ± 0.77	9.41 ± 3.59	15.45 ± 0.28	13.54 ± 8.54	29.97 ± 0.15	15.02 ± 8.97	14.87 ± 8.67	30.00 ± 0.00	14.44 ± 8.99	15.04 ± 8.67	30.00 ± 0.00	14.37 ± 8.98
8	0.45 ± 0.39	4.49 ± 1.78	8.34 ± 0.33	9.39 ± 6.90	29.51 ± 1.33	17.50 ± 8.52	14.31 ± 8.64	30.00 ± 0.04	14.60 ± 9.03	15.01 ± 8.67	30.00 ± 0.01	14.32 ± 9.02
*P* value	*P <* 0.05	*P <* 0.05	*P <* 0.05	*P <* 0.05	*P <* 0.05	*P <* 0.05	NS	NS	NS	－	－	－

NS: not statistically significant (*P* > 0.05).

### The relationship between ET opening degree and middle ear pressure equilibrium

CFD simulations demonstrated that partial ET opening (30%) achieved nearly identical middle ear (tympanic cavity, mastoid antrum, and air cells) pressure equilibration compared with full opening (100%) in two of eight ears (25%). Notably, six ears (75%) required only 50% ET opening to reach full pressure equilibration across all phases—pressurization, stabilization, and depressurization—as summarized in Tables 2–4.

Our study further revealed that in the first two ears, when the ET was partially opened (30%), middle ear cavity pressure returned to zero or exhibited slight negative pressure after the decompression phase. In the remaining six ears, similar outcomes were observed with 50% ET opening. However, when ET opening was restricted to 10%, middle ear cavity pressure remained elevated following decompression. To further investigate this phenomenon, additional measurements of middle ear cavity pressure were performed at 20% ET opening in the same specimens where 50% opening produced results comparable to full opening (100%). The 20% patency group still demonstrated incomplete pressure recovery, reinforcing that ≥50% ET patency represents the threshold for effective middle ear pressure equilibration—consistent with the 75% ear proportion observed in our cohort. These results follow the same trend illustrated in Fig 2B.

## Discussion

This study developed an anatomically accurate CFD model of the ET–middle ear system based on HRCT scans and systematically analyzed pressure dynamics under different ET patency levels (10%, 30%, 50%, and 100%), validated using in vivo TMM data. The principal finding is that partial ET opening (≥50% patency) achieves pressure equilibration in the middle ear equivalent to full opening (100%), whereas patency below 50%—particularly at 10%—results in incomplete pressure transmission and residual elevated pressure. This finding bridges an important gap in the literature by quantitatively defining the threshold for effective ET ventilation and provides new insights into middle ear physiology and clinical management of ETD.

The simulated pressure dynamics revealed a clear dose–response relationship between ET patency and middle ear pressure transmission. At 10% patency, extensive low-pressure zones persisted within the ET and adjacent middle ear structures (Fig 4), with pronounced pressure gradients impeding effective equilibration—consistent with the elevated residual pressure observed in the 10% patency group (Fig 2B; Tables 2–4). As patency increased to 20–30%, these pressure gradients gradually diminished, yet equilibration remained incomplete, as evidenced by statistically significant differences in pressure indicators compared with the 50% and 100% patency conditions. Once patency reached 50%, the entire ET–middle ear system exhibited a uniform pressure distribution (warm color regions in the contour plots; Fig 4), with no statistically significant differences in pressurization rate, plateau pressure, or depressurization characteristics relative to 100% patency. These findings indicate that a 50% patency threshold is sufficient to overcome the rate-limiting resistance of the isthmus to airflow, allowing complete pressure equilibration throughout the system.

Crucially, the persistent pressure gradient from the ET lumen toward the middle ear during pressurization (ET pressure > middle ear pressure) supports the hypothesis that the ET functions as a pressure-driven valve rather than a passive conduit [9]. The near-complete equilibration observed during stabilization, along with the reversal of airflow during depressurization, further validates the model’s ability to reproduce physiological pressure cycling.

The pressure dynamics observed across the three distinct phases—pressurization, stabilization, and depressurization (Fig 3)—demonstrate several key physiological characteristics of ET function. In all tested ears, as inlet pressure continued to increase, the airflow pressure at the narrowest segment of the ET (the isthmus) exhibited a marked decline. This observation closely aligns with the findings reported by Tang Yuanyuan et al. [7]. The identification of the isthmus as the critical region where pressure reaches its minimum—due to the Venturi effect (peak velocity at the smallest cross-sectional area)—offers a biomechanical explanation for the heightened vulnerability of this anatomical site to pathological changes. This interpretation is consistent with clinical evidence indicating that ET dysfunction commonly involves the narrow osseous–cartilaginous transition region formed by the isthmus [10].

Previous studies on ET function have primarily employed a binary “open/closed” framework, with limited attention to the functional implications of partial opening. For example, in a systematic review, Smith et al. [11] defined pathological ET states using a binary “open/closed” classification, directly categorizing ET dysfunction into obstructive and patulous types. McDonald et al. [8] obtained continuous imaging sequences of healthy participants during swallowing and, through analysis of these sequential frames, concluded that effective ET ventilation does not require complete opening. Zhu et al. [12] described the dynamic behavior of the ET, characterized by “sequential opening and multistage pressure changes,” confirming that middle ear ventilation results from the combined action of the ET and associated structures such as the mastoid air cells. Angelstedt et al. [13] reported that elevating the body by 20 degrees reduced average ET airflow by one-third, whereas in the supine position, airflow decreased by two-thirds. A renewed understanding of the ET opening mechanism enhances comprehension of ETD and supports the development of more precise surgical and therapeutic interventions. The most notable finding of this study is that 50% ET patency achieved complete pressure equilibration in 75% of ears (Tables 2–4; Fig 2B), while even a 30% opening was sufficient in 25% of cases. This observation challenges the conventional binary model that classifies ET function simply as “open” or “closed,” demonstrating instead that the system possesses substantial functional reserve capacity in pressure regulation. It also implies possible interindividual variations in the efficiency of partial ET function. The results strongly suggest that in many individuals, the ET can perform its primary pressure-regulating role effectively without requiring full dilation. Approximately 50% patency appears sufficient to ensure adequate ventilation and pressure balance in a considerable proportion of cases. This finding holds important implications for understanding ETD pathophysiology: symptom onset may not necessarily result from complete obstruction but rather from failure to achieve or sustain the critical partial opening threshold during swallowing or other physiological activities. Furthermore, the data indicate that therapeutic approaches aimed at achieving “complete” ET opening—such as balloon dilation—may be unnecessarily aggressive. Targeting a functional opening threshold (approximately 50% patency) could maintain therapeutic efficacy while minimizing the risk of procedure-related complications.

## Conclusion

This CFD study quantitatively demonstrates that effective middle ear pressure equilibration can be achieved with partial ET opening (30–50%) in most cases (75% of tested ears). The findings provide the first quantitative evidence that partial opening is sufficient to achieve pressure balance, thereby offering a theoretical foundation for optimizing ETD treatment and contributing new insights into middle ear physiology and its responses under pathological conditions.

## Limitations and recommendations

The primary limitation of this study lies in its small sample size, which restricts the generalizability of the results. An expanded cohort is required in future investigations to validate these findings and strengthen their correlation with clinical outcomes. Additionally, the current CFD model assumes a rigid ET structure. Subsequent studies should incorporate the biomechanical properties of ET tissues to enable a more physiologically realistic representation of tube dynamics and function.
